# Mechanisms, Biomarkers, and Therapeutic Interventions of Neuroplasticity After Ischemic Stroke—A Scoping Review

**DOI:** 10.3390/brainsci16080784

**Published:** 2026-07-25

**Authors:** Pingping Yang, Dan Xie, Song Wang, Yingying Zhao, Yongbo Zhang

**Affiliations:** Department of Neurology, Beijing Friendship Hospital, Capital Medical University, Beijing 100050, China; pingyxy@sina.com (P.Y.);

**Keywords:** neuroplasticity, ischemic stroke, mechanisms, biomarkers, rehabilitation, therapeutic interventions

## Abstract

**Highlights:**

**What are the main findings?**

- A complex network of interrelated biological pathways mediates neuroplasticity following ischemic stroke. Preclinical studies have confirmed that key plastic changes include enhanced synaptic function, remodeling of dendrites and axons, restored communication between the two cerebral hemispheres, increased neurogenesis, and functional rearrangement of neural circuits, as well as compensatory neural activation in peri-infarct tissue and distant brain regions.
- Clinical studies on neuroplasticity biomarkers mainly focus on electrophysiology and neuroimaging, while most molecular and genetic research remains in the exploratory stage. Uniform evaluation criteria are still absent, and their predictive values have not been fully verified.

**What are the implications of the main findings?**

- Most studies on neuroplastic mechanisms remain at the preclinical stage, and relevant clinical evidence is still insufficient. This review proposes potential research directions for further exploring the mechanisms underlying functional recovery after ischemic stroke.
- This study systematically summarizes graded neuroplasticity mechanisms and assesses existing research limitations. It provides a theoretical reference and new research ideas for further exploring effective biomarkers, as well as promoting the clinical translation of stroke rehabilitation intervention strategies.

**Abstract:**

**Background**: Stroke remains a significant cause of persistent long-term disability globally. Post-stroke neuroplasticity is critical for neurological functional recovery and reducing disability. Nevertheless, the existing scoping reviews rarely systematically integrate its intrinsic mechanisms, predictive biomarkers, and actionable intervention strategies. This scoping review aims to map the current research landscape, synthesize the core research findings, and identify existing research gaps in this field. **Methods**: This scoping review was conducted following the PRISMA-ScR guidelines. Eligible studies published between 31 January 2021, and 31 January 2026, were retrieved from three major mainstream electronic databases: PubMed, Scopus, and Web of Science. All retrieved evidence was synthesized via narrative approach, focusing on core research findings concerning post-stroke neuroplasticity mechanisms, biomarkers, and therapeutic interventions. **Results**: The existing research on neuroplastic mechanisms following ischemic stroke is predominantly derived from in vitro and animal studies, which have collectively demonstrated multiple core adaptive alterations, including enhanced synaptic plasticity, dendritic and axonal structural remodeling, restored interhemispheric connectivity, endogenous neurogenesis, and functional reorganization of neural networks. In contrast, mechanistic investigations in human stroke patients primarily highlight compensatory activation within peri-infarct tissues and functionally remote brain regions. The relevant clinical biomarkers are mainly imaging and electrophysiological indicators. Research on therapeutic interventions has mainly focused on rehabilitation treatments such as non-pharmacological magnetic stimulation. **Conclusions**: This scoping review synthesized the current body of evidence on post-ischemic stroke neuroplasticity and identified critical research gaps, particularly regarding multimodal biomarkers and their translational relevance. The present findings confirm that neuroplastic alterations after ischemic stroke are regulated by multiple pathways, among which those involved in synaptic structure, dendrites, and neural network connections exert pivotal effects. Nevertheless, most existing investigations remain confined to in vitro experiments and animal models. The identification of neuroplasticity biomarkers has opened up new avenues for the development of targeted stroke therapies and offers promising prospects for rehabilitative treatment of stroke patients.

## 1. Introduction

Stroke remains a leading global cause of disability and represents the primary neurological disorder contributing to disability-adjusted life years (DALYs). Epidemiological investigations have found that about 75% of stroke survivors are left with residual sequelae and approximately 40% of patients are left with severe disabilities. Stroke causes tremendous economic losses and physical–mental suffering for society and families [1]. Ischemic stroke is the most common subtype of stroke. The primary treatment for acute ischemic stroke is intravenous thrombolysis or combined endovascular thrombectomy. However, while thrombolytic therapy can partially restore blood flow, its narrow therapeutic time window and risk of hemorrhage limit its widespread application. There is still a lack of effective strategies to promote functional recovery after a stroke [2,3].

Neural plasticity refers to the nervous system’s ability to respond to damage or new signals, and to modify, regulate, and regenerate adaptations [4]. Although these changes cannot achieve significant recovery after brain injury, they can still contribute to improved outcomes in stroke patients. Current research on post-ischemic stroke neuroplasticity is fragmented: there are numerous separate reports on molecular mechanisms, various biomarkers, rehabilitation, and neuro-modulation interventions [5,6,7], but a comprehensive synthesis of the overall evidence is lacking.

Several literature reviews have examined neuroplasticity after ischemic stroke. However, existing reviews focus on different discrete aspects of neuroplasticity: some on its underlying mechanisms [8,9,10,11], others on its biomarkers [12,13], and still others on its intervention strategies [14,15]. Traditional systematic reviews typically focus on only one or two of these research directions [4,16,17]. Furthermore, current review reports do not properly categorize plasticity-related mechanism studies as either consolidated evidence or exploratory research directions. They fail to distinguish plasticity biomarkers by developmental stage: clinically validated mature biomarkers, preliminarily verified potential biomarkers, and experimentally exploratory biomarkers. Nor do they differentiate between preclinical and clinical studies of neuroplasticity interventions.

Therefore, a scoping review was conducted to comprehensively map the existing research. It summarizes the mechanisms of neuroplasticity and recent research progress on post-stroke plasticity biomarkers and interventions to improve the prognosis of stroke patients from a new perspective. Specifically, we identify current research hotspots, highlight discrepancies among different studies, classify the current evidence on plasticity mechanisms, biomarkers, and intervention studies, address gaps overlooked in existing reviews, and identify future research directions.

### Aim and Objectives

Aim: To systematically map and rigorously evaluate the existing evidence on the mechanisms, biomarkers, and interventions for neuroplasticity after ischemic stroke.

Objectives:

1. What are the cellular, molecular, and brain-network mechanisms underlying neuroplasticity after ischemic stroke?
2. What biomarkers (molecular, electrophysiological, and imaging) can reflect neuroplastic changes and predict functional recovery?
3. What intervention strategies (rehabilitation, pharmacological therapy, and neuromodulation) are applied to modulate post-stroke neuroplasticity?

## 2. Methods

This scoping review was conducted following the Preferred Reporting Items for Systematic Reviews and Meta-Analyses Extension for Scoping Reviews (PRISMA-ScR) guidelines [18], and the completed checklist is provided in Supplementary Table S1. The protocol has been registered with the Open Science Framework (https://osf.io/78zxd, accessed on 10 July 2026).

### 2.1. Eligibility Criteria

This scoping review followed the Population–Concept–Context (PCC) framework to define the eligibility criteria.

Population: Patients diagnosed with ischemic stroke, aged ≥18 years, covering acute (≤7 d), subacute (8 d–6 months), and chronic phases (>6 months).

Concept: Studies that explicitly address at least one of the following post-ischemic stroke neuroplasticity-related topics:

- Mechanisms of neuroplasticity;
- Predictive or evaluative biomarkers for neuroplasticity;
- Pharmacological or non-pharmacological interventions targeting neuroplasticity.

Context:

- Study type: Peer-reviewed original clinical research (e.g., RCTs, observational studies, clinical trials, and prospective/retrospective cohort studies);
- Language: English;
- Publication date: Between 31 January 2021, and 31 January 2026.

The reasons for only including English literature are as follows: (1) English is the universal academic language in the field of neuroscience, and high-quality basic and clinical studies are mainly published in English peer-reviewed journals; (2) the use of a single language ensures unified research terminology, standardized experimental protocols, and consistent evidence interpretation and conclusions by avoiding potential language translation deviations; and (3) when combined with a focus on peer-reviewed literature, it guarantees the academic rigor and international representativeness of the scoping review. However, we still list this as a limitation in Section 4.3 for objective and transparent academic reporting.

The exclusion criteria were as follows: studies focusing exclusively on animal models, in vitro experiments, or non-ischemic stroke populations (e.g., hemorrhagic stroke and transient ischemic attack); studies without an explicit focus on post-ischemic stroke neuroplasticity; research on clinical or mortality outcomes that does not include mechanistic, biomarker, or plasticity-related analyses; non-English publications; reviews, meta-analyses, commentaries, editorials, letters, conference abstracts, case reports, and non-empirical papers; and studies published outside the 31 January 2021 to 31 January 2026 time period.

Although animal experiments, in vitro studies, and review articles were formally excluded from formal inclusion, to ensure the comprehensiveness of this scoping review, we still collated and summarized the core findings from these types of literature. Citations of animal experiments and in vitro studies serve to provide a biological background and elaborate underlying neurobiological mechanisms. Given the limited number of current clinical studies investigating the relevant mechanisms and biomarkers, referencing such preclinical evidence also helps identify research gaps and provides implications for future directions of clinical research.

### 2.2. Information Sources and Search Strategy

Literature searches were performed across three major databases: PubMed, Scopus, and Web of Science. The search strategy combined Medical Subject Headings (MeSHs) and keywords, including neuroplasticity, ischemic stroke, rehabilitation, mechanisms, biomarkers, and interventions, which were combined using the Boolean operators AND and OR.

This study adopted a comprehensive narrative synthesis method to systematically collate and summarize the current evidence on the core mechanisms, biomarkers, and intervention strategies for post-stroke neuroplasticity. Narrative reviews were also consulted, and citation screening was performed to identify additional eligible studies.

The initial search strategy was first established for PubMed and subsequently adapted for the other databases, complying with the unique controlled vocabularies and syntax rules of each respective database. The full PubMed search strategy is detailed in Supplementary Table S2. Searches were performed in the title and abstract fields. All bibliographic records were managed using Zotero (Version 9.0.6).

### 2.3. Selection of Sources of Evidence

**Figure 1:**
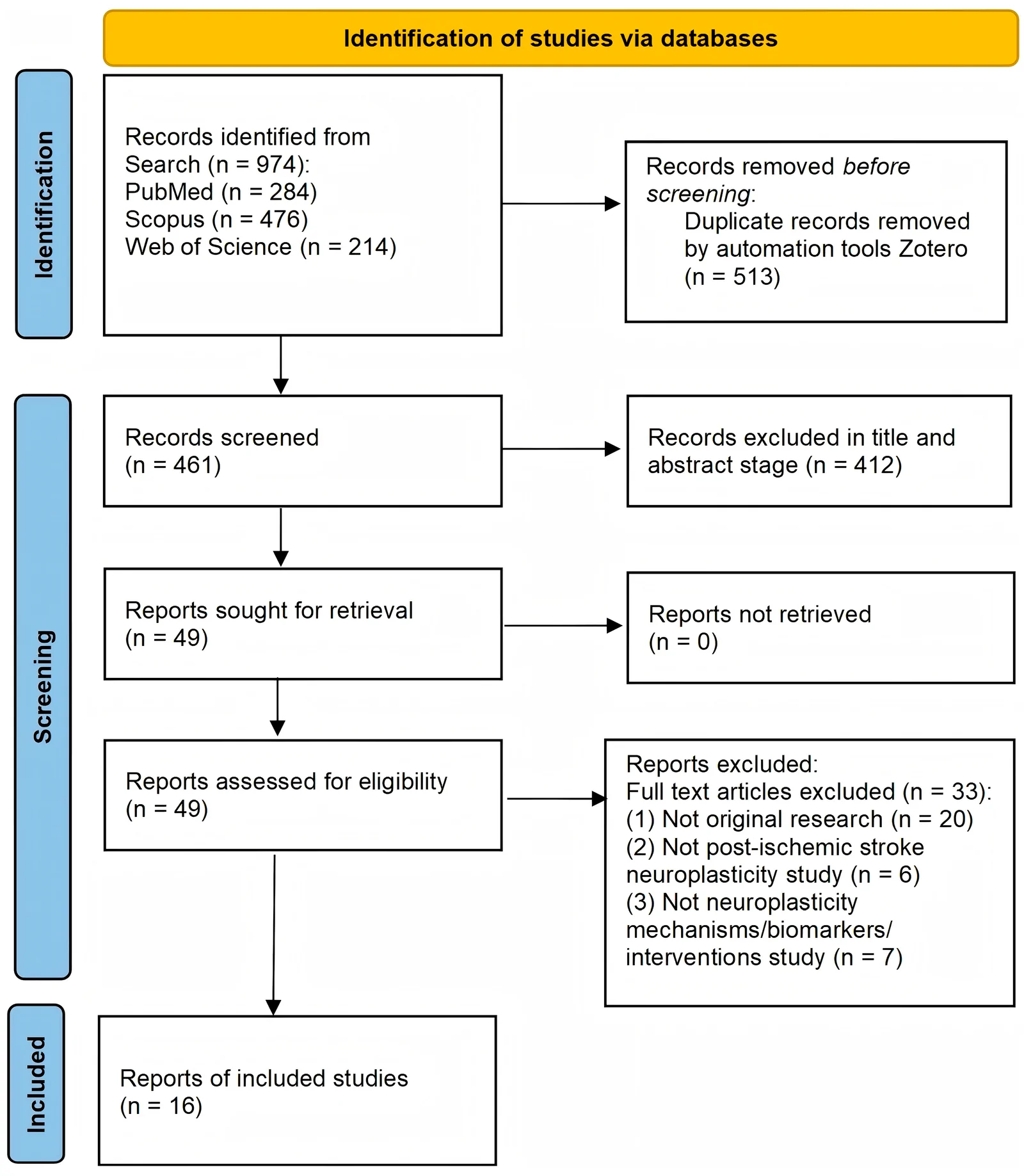
PRISMA-ScR flow diagram of the study selection process.

A total of 974 records were obtained from the initial search and were imported into Zotero for screening. After duplicates were removed, 461 unique records were independently screened by two researchers (P.Y. and Y.Z.) based on titles and abstracts, with all exclusion reasons fully documented. Any discrepancies were resolved through regular group discussions, and unresolved disputes were adjudicated by a third researcher (D.X.). Eligibility assessment was then conducted on full-text articles, and relevant studies were finally included in the comprehensive analysis. The detailed study selection process is shown in the PRISMA flow diagram (Figure 1).

### 2.4. Data Extraction

All researchers independently completed data extraction using a self-designed form based on the review objectives and eligibility criteria. The extracted contents were author and year, country, study design, research subjects (stroke patients or animal models), total sample size, research domain (mechanisms, biomarkers, or interventions), and key findings.

### 2.5. Critical Appraisal of Individual Sources of Evidence

This scoping review focuses on mapping the current research landscape, identifying key themes, and highlighting knowledge gaps, rather than evaluating intervention efficacy or methodological quality. Due to the broad scope and high heterogeneity of included study designs and outcomes, no universal quality assessment tool was applicable to this review. Accordingly, we present our findings narratively and acknowledge that the included studies vary in their levels of evidence.

### 2.6. Data Analysis and Synthesis

The analysis comprised descriptive numerical synthesis of study characteristics and a narrative synthesis focusing on neuroplasticity mechanisms, biomarkers, intervention modalities, methodology, and sample sizes. All data were tabulated to illustrate overall research patterns and the heterogeneity across the included studies.

## 3. Results

### 3.1. Search Results

The PRISMA flow diagram (Figure 1) outlines the selection process. From 974 initial records, 513 duplicates were removed. Screening of 461 titles and abstracts excluded 412 records. Full-text assessment of 49 articles led to the exclusion of 33, resulting in 16 primary studies included in the final synthesis.

### 3.2. Characteristics of Included Studies

The characteristics of the 16 included studies are summarized in Table 1. Among them, only three studies investigated mechanisms of post-stroke neuroplasticity [6,7,19], with only one being a multicenter study [20]; four studies investigated biomarkers of post-stroke neuroplasticity [19,21,22]; interventions targeting neuroplasticity were the most prevalent [23,24,25,26]. Non-pharmacological therapies were employed for neuroplasticity treatment in stroke patients, many of which involved brain stimulation [20,27]. The study designs were primarily controlled trials or observational studies, and several had small sample sizes [28,29,30].

### 3.3. Mechanisms of Post-Ischemic Stroke Neuroplasticity

The pathological progression following a stroke is characterized by four consecutive stages: hyperacute (0–24 h), acute (1–7 days), subacute (1 week to 3 months), and chronic (exceeding 3 months). Recent research by Cirillo and Beker et al. found that the subacute phase is a critical period for stroke recovery and during this period, neural plasticity reaches its peak [31,32,33,34]. Many studies have elaborated the main mechanisms underlying post-stroke neuroplasticity. However, most of these studies were conducted in animal models and in vitro, as shown in Supplementary Table S1.

**Figure 2:**
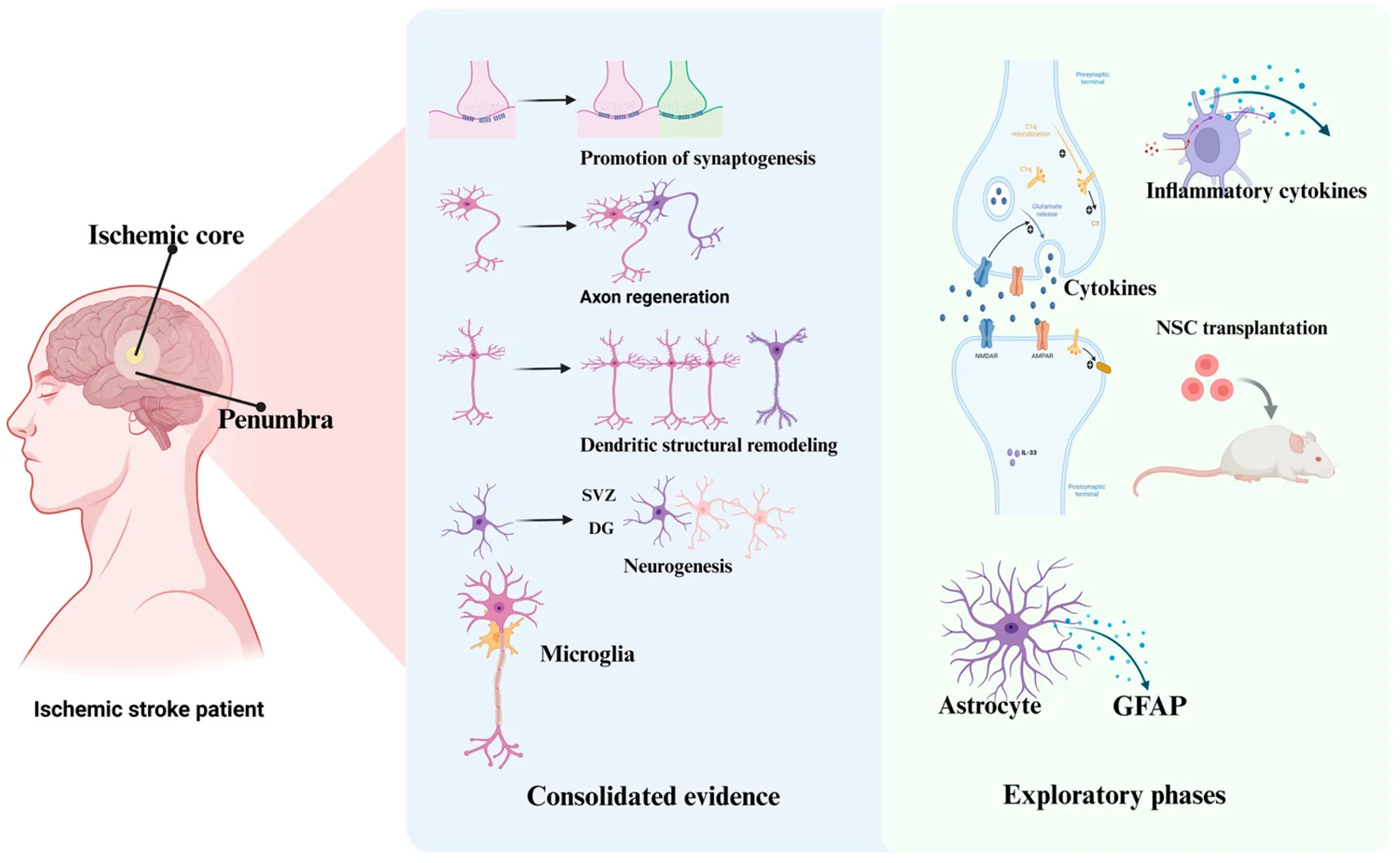
Potential mechanisms of post-ischemic stroke neuroplasticity. The schematic displays the ischemic core and penumbra of stroke lesions, divides neuroplastic research findings into consolidated evidence (neurogenesis, axonal regeneration, dendritic remodeling, synaptogenesis, microglia regulation) and exploratory research directions (inflammatory cytokines, NSC transplantation, GFAP + astrocyte activation). These mechanisms are derived from animal studies and in vitro research.

One post-stroke neuroplasticity mechanism is to promote synapse formation and axon regeneration, allowing new dendrites, axons, and synapses to grow from existing cell bodies to restore damaged central nervous system cells and enhance the connections between the cerebral hemispheres [4,35,36,37]. Another mechanism is the massive generation of new neurons in two neurogenic regions, the subventricular zone (SVZ) and the dentate gyrus (DG), leading to brain functional reorganization and neural compensation outside the infarcted tissue [38]. Figure 2 systematically summarizes the neuroplastic mechanisms reported in the current literature and sorts out mature and exploratory research directions.

#### 3.3.1. Molecular and Cellular Mechanisms

Accumulating evidence from animal studies has revealed that the predominant molecular mechanisms after stroke involve promoting dendritic arborization, as well as enabling axonal and synaptic regeneration based on surviving neuronal somata, thereby facilitating the repair and reconstruction of impaired central neural circuits [35]. Adaptive plasticity pathways include brain-derived neurotrophic factor (BDNF)-mediated synaptic remodeling, activation of the PI3K/Akt signaling pathway, coordinated repair of neurovascular units, and activating the bidirectional regulatory effects of microglia [10,16,39]. Rodent experiments demonstrated that neurons around cerebral infarcts generate new axons that extend toward adjacent cortices and form functional synapses. Axons from neurons in contralateral brain regions also project to bilateral denervated areas [10]. The corpus callosum mediates interhemispheric neural communication, balances bilateral cortical excitability, and participates in axonal remodeling and compensatory neural circuit reconstruction after ischemic stroke. Aberrant neural connections in the unaffected cerebral hemisphere hinder functional recovery [40]. Abnormal callosal pathways suppress cortical activity of paretic limbs, and blocking newly formed connections is also detrimental to rehabilitation [41,42]. Endogenous neurogenesis is tightly correlated with synaptogenesis [8,24,25,26] and involves the establishment of presynaptic neurotransmitter release sites [24] and postsynaptic signal reception domains, and precise structural matching of synaptic junctions [10,43].

#### 3.3.2. Brain Network Reorganization

After ischemic stroke, the neuroplastic alterations occur in distinct phases. In the acute phase (within 1 week), neurons surrounding the infarct core undergo transient suppression of neuronal excitability [44]. The subacute phase (8 days to 6 months) represents the peak period of brain plasticity, during which the ipsilateral motor cortex is gradually activated and the interhemispheric inhibitory balance undergoes dynamic reconstruction. In the chronic phase (after 6 months), long-term remodeling occurs in distant brain networks including the default mode and salience networks [45]. Excessive activation of the contralateral cerebral cortex at this stage tends to induce maladaptive neuroplasticity, which is unfavorable to neurological functional recovery [43,44]. Multiple clinical trials have confirmed that neural stem cell (NSC) replacement therapy can produce positive therapeutic effects in stroke patients [45,46]. Newly formed cortical neural circuits can be detected as early as three weeks after ischemia, which is closely related to functional recovery [43]. In the subventricular zone, neuroblasts are generated and migrate along the rostral migratory stream to the olfactory bulb, where they differentiate into granule cells and periglomerular interneurons involved in the plasticity processes of olfactory learning. In the dentate gyrus, stem cells produce neuronal precursors, which mature and generate new granule neurons [47]. Studies have found that the expression of glial fibrillary acidic protein (GFAP) [48], Ang1, stromal cell-derived factor-1α (SDF1α) [7,49], and inflammatory factors [19,50] influences the occurrence of neuroplasticity after stroke. In the V-SVZ of the lateral ventricle, most GFAP-positive adult neural stem cells express Epac2 proteins, which are involved in adult neurogenesis in the ventricular–subventricular zone and subgranular zone [51].

### 3.4. Biomarkers of Post-Ischemic Stroke Plasticity

The research on biomarkers of post-ischemic stroke plasticity has mainly focused on a few types of biomarkers. One type is imaging and electrophysiological-related biomarkers, including resting-state functional magnetic resonance imaging (rs-fMRI) [17,48,52], transcranial magnetic stimulation (TMS) [53,54], magnetoencephalography (MEG) [55,56,57], and electroencephalography (EEG) [58,59,60] biomarkers. Different abnormal manifestations during various post-stroke rehabilitation phases can be observed in alpha, beta, and gamma waves, accompanied by obvious changes in the functional modulation of alpha frequency bands [61]. Studies on ischemic injury models of the primary motor cortex have revealed that transient dynamic reorganization of brain networks occurs during the early stage of functional recovery after stroke during sleep [62]. Among the included studies in this scoping review, four focused on tDCS [20,23,26,27], two used rs-fMRI [21,63], and one adopted EEG [21] to identify biomarkers of plasticity. These three are the primary modalities currently applied in clinical practice.

Another type of biomarker associated with stroke is molecular genetic markers such as neurotrophic factors [64,65,66], inflammatory factors, miRNAs, and S100B protein [11,12,14,66]. These include BDNF [64,65,67], NGF, and inflammatory factors [68,69]. In our literature searches, we found numerous recent studies on microglia [70]. Genetic markers mainly include microRNAs [71,72,73,74], CCR5 and CXCR4 [75,76], and GRM1 [77,78,79]. Altered miRNA expression profiles in blood and brain tissues of patients with acute ischemic stroke suggest their utility in stroke detection, subclassification, and outcome prediction [49,80,81]. S100B is primarily expressed in Schwann cells and astrocytes in the CNS; significantly elevated peripheral S100B levels have been detected in patient and animal models after ischemic stroke [82,83,84]. GRM1 is a critical glutamate receptor that is a key regulator of synaptic plasticity and has been implicated in neuropsychiatric disorders and tumor biology [77,78,79].

**Figure 3:**
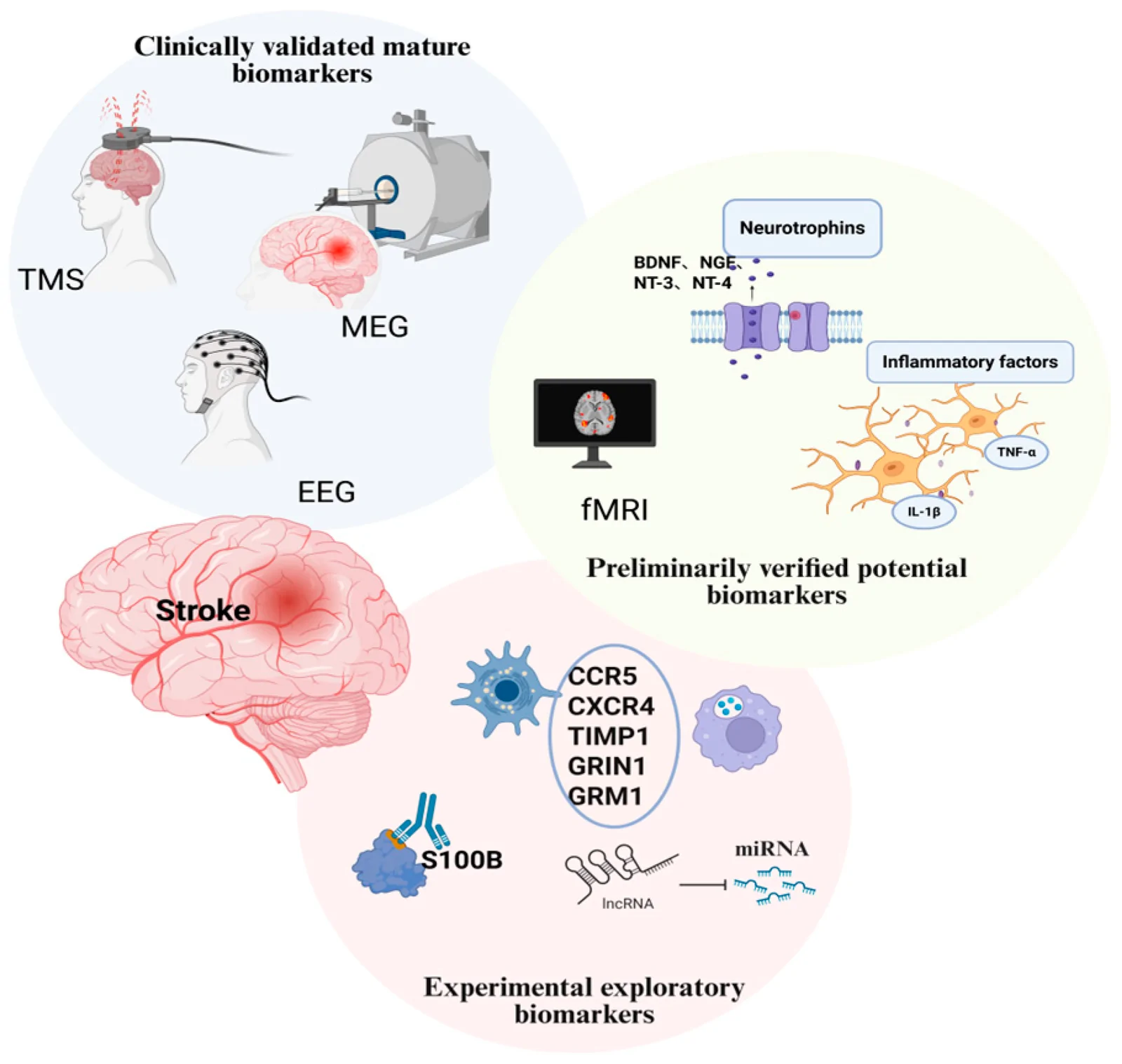
Classification of biomarkers for evaluating neuroplasticity after ischemic stroke. Based on research maturity, relevant biomarkers are divided into three categories: clinically validated mature biomarkers including electrophysiological and neuroimaging detection modalities (EEG, MEG, TMS, fMRI); preliminarily verified potential biomarkers covering neurotrophins and inflammatory factors; and experimental exploratory biomarkers consisting of protein molecules (S100B, CCR5, CXCR4, TIMP1, GRIN1, GRM1) and microRNAs.

Among the studies included in this review, one is related to immune-inflammatory activation [22] and another focused on serum TRAIL and adropin levels [5]. All other relevant molecular markers have only been studied in vitro or in animal models (Supplementary Table S3). A comprehensive summary of the current state of biomarker research is provided in Figure 3.

### 3.5. Pharmacological and Non-Pharmacological Interventions for Post-Ischemic Stroke Plasticity

Post-ischemic stroke plasticity-oriented interventions can be divided into pharmacological and non-pharmacological interventions. In the discussion below, the interventions are categorized into clinically established interventions and experimental preclinical interventions based on their degree of clinical validation and translational maturity.

#### 3.5.1. Clinically Established Interventions

Among the pharmacological interventions, only monoaminergic agents and several traditional Chinese patent medicines (e.g., Yindan Xinnaotong Soft Capsule [65], Neural Fuyuan Formula [85], and Zhongfeng Xingnao [86]) have been applied clinically. However, it should be noted that monoaminergic agents are primarily prescribed for post-stroke depression rather than for the direct improvement of neurological deficits caused by ischemic stroke [87,88] and although the traditional Chinese patent medicines are clinically available, their neuroplasticity-modulating mechanisms—which mainly involved the CREB/BDNF, BDNF/TrkB, and SIRT1/Nrf2/HO-1 signaling pathways—have been predominantly validated in animal models, with limited clinical mechanistic evidence [65,85,86].

Regarding non-pharmacological approaches, some physical exercise regimens, including general physical training, aerobic walking, social dancing, and mirror therapy, have accumulated preliminary clinical evidence [24,63,89,90]. Preclinical and clinical studies have demonstrated that these approaches promote neural plasticity, likely by elevating peripheral neurotrophic factors and anti-inflammatory mediators, thereby facilitating post-stroke functional recovery. Furthermore, non-invasive brain stimulation techniques, including transcranial magnetic stimulation (TMS) and transcranial direct current stimulation (tDCS) [12,91,92], have been preliminarily confirmed to modulate neuroplasticity for stroke rehabilitation, serving as mainstream clinically applicable non-pharmacological interventions [20,26,27,28].

#### 3.5.2. Experimental Preclinical Interventions (Under Investigation)

Most pharmacological interventions remain at the preclinical exploratory stage without standardized clinical implementation. These experimental candidates include the biological agent annexin A6 [5,93,94], targeted gene therapies [79], and chemical preparations such as coenzyme Q10 [94] and methylphenidate [86]. Despite their promising effects on regulating post-stroke neuroplasticity in basic studies, the evidence for their efficacy is currently restricted to preclinical findings, and their clinical efficacy and safety in ischemic stroke patients require further validation.

Multiple innovative non-pharmacological therapies are also experimental and preclinically limited, including brain–computer interface intervention [15], robot-assisted rehabilitation [29], virtual reality therapy [29,30], and cellular therapy [15,88,95]. Preclinical studies have indicated that cellular therapy can enhance neuroplastic function and improve stroke prognosis [15,96,97]. In addition, auditory, visual, and electromagnetic brain stimulation paradigms, as well as transcranial light stimulation, have shown favorable effects on enhancing neuroplasticity and neurological recovery in preclinical models of chronic cerebral ischemia [95,98].

In summary, although these emerging interventions possess considerable potential for alleviating post-stroke functional impairments, most supportive evidence is derived from preclinical studies. Currently, clinical strategies for precise regulation of stroke-related neuroplasticity remain insufficient, highlighting the necessity of high-quality clinical studies to validate the translational value of existing experimental interventions.

### 3.6. Comparison of This Scoping Review and Previous Reviews

After reviewing previously published reviews, we found that most existing studies on the mechanisms, biomarkers, and therapeutic interventions for post-stroke neuroplasticity concentrate on a single research domain, and the majority are traditional narrative reviews (Supplementary Table S4). In our literature search, only two scoping reviews [12,17] and one systematic review with meta-analysis [13] were retrieved. Accordingly, extant reviews are inconsistent in methodological quality, lacking robust synthesized evidence and having limited reference value for clinical practice and academic research. In contrast, the present study was conducted as a rigorous scoping review following standard methodological guidelines. We performed comprehensive literature searches covering three core aspects of neuroplasticity research: intrinsic mechanisms, candidate biomarkers, and therapeutic interventions.

Furthermore, this review systematically collated recent research advances, outlined the research hotspots, clarified the existing limitations, and highlighted unaddressed research gaps in this field. It is expected to provide more holistic, standardized, and reliable evidence for future basic experimental studies and clinical investigations focusing on post-stroke neuroplasticity.

### 3.7. Distribution and Inconsistency of Current Evidence

Despite growing interest in post-stroke neuroplasticity, several critical gaps persist in the current evidence base. First, most studies have focused on the acute [5,7,22] and subacute phases [20,26], with a notable scarcity of investigations addressing long-term chronic plasticity [30,99,100]. Furthermore, some studies did not clearly specify the stroke phase under examination [6,19,21,24], limiting the comparability and applicability of the findings. Second, most studies on core biomarkers only identified a single biomarker, and were mostly conducted in in vitro and animal models, leaving research gaps in this field. Sprague Dawley rats are widely used in biomarker modeling studies, and some studies fail to specify the source of the Sprague Dawley rats. Obvious discrepancies exist between research models, making it difficult to formulate standardized judgment criteria. Third, there is a lack of research on individualized drug and neuromodulation combination therapy, resulting in a major gap in the development of targeted rehabilitation treatment plans precisely tailored to specific patients. Lastly, the sample sizes in the published clinical studies are insufficient, which may lead to biases and fail to accurately reflect the efficacy of plasticity interventions.

## 4. Discussion

### 4.1. Main Findings and Controversies of Neuroplasticity

Based on the existing research evidence strength, regulatory effects, research maturity, and clinical correlations, we classified post-stroke neuroplasticity mechanisms into consolidated evidence (neurogenesis, axonal regeneration, dendritic remodeling, synaptogenesis, microglia regulation), exploratory research directions (inflammatory cytokines, NSC transplantation, GFAP + astrocyte activation), and potential emerging mechanisms (such as epigenetic regulation). These mechanisms are mostly derived from animal experiments and in vitro studies.

Neuroplastic reorganization following ischemic stroke is modulated by sophisticated multi-level spatiotemporal regulatory mechanisms, which constitute the biological basis for post-injury neurological functional rehabilitation. Including robust dendritic arborization, axonal sprouting, and regeneration of synapses anchored to viable residual neuronal somata, which collectively facilitate the repair and reconstruction of impaired central neural circuits. Multiple core signaling pathways and cellular components synergistically mediate these plastic changes. However, the clinical value of endogenous neurogenesis after ischemic stroke remains highly controversial, and human clinical evidence is still insufficient.

Accumulating studies have focused on microglia, while the dual roles of glial cells remain highly controversial. Traditionally, anti-inflammatory M2-type microglia are considered to clear cellular debris and secrete neurotrophic factors to promote neural remodeling [4,43,71]. Nevertheless, emerging evidence has challenged this oversimplified viewpoint, confirming that pro-inflammatory M1-type microglia also participate in synaptic pruning and neural circuit reconstruction [8,99,101].

Regarding biomarkers of post-stroke neuroplasticity, those validated in clinical trials are predominantly derived from non-invasive imaging and electrophysiological techniques. Rs-fMRI is one of the recommended techniques for evaluating human neural plasticity and is commonly used to examine resting-state functional networks [13,14,17,48,52,102,103]. TMS technology uses a magnetic coil close to the scalp to generate a magnetic field that induces currents in specific brain regions [53,54,104]. Research suggests that transcranial direct current stimulation (tDCS) may mediate neuromodulation partly by upregulating the expression of neuroplasticity-related genes in the frontal cortex [23,105,106,107]. In stroke biomarker research, MEG and EEG are primarily applied to predict motor recovery, assess the reorganization of language networks, and monitor cognitive impairment [55,56,57,108]. EEG, a non-invasive neuroelectrophysiological tool with high temporal resolution, is progressively evolving from a conventional diagnostic instrument into a key modality for assessing neuroplasticity [21,25,101]. Electrophysiological and functional imaging biomarkers all suffer from high research heterogeneity, poor repeatability, and cannot effectively distinguish spontaneous recovery from intervention-induced neuroplastic changes.

In studies concerning molecular and genetic biomarkers, we found that BDNF is the most extensively investigated and highly controversial biomarker linked to neuroplasticity. Serum BDNF levels show inconsistent changing trends after stroke [66]. Rehabilitation and neuromodulation cannot stably and durably regulate peripheral BDNF expression [64,85]. BDNF gene polymorphism leads to obvious individual differences in rehabilitation response, which restricts its application as a stratified predictive marker for personalized rehabilitation. Key signaling pathways including BDNF/PI3K/Akt, BDNF/mTORC1/SIRT1, and BDNF/Trkβ show dose-dependent and time-dependent bidirectional regulatory effects, further increasing clinical application difficulties [25,85].

In the research field of interhemispheric interaction of neuroplasticity after ischemic stroke, two mainstream hypotheses have been put forward to interpret the mechanism of interhemispheric functional imbalance. The contralesional overactivation hypothesis proposes that excessive neural excitation in the intact unaffected hemisphere should be restrained, whereas the ipsilesional hypoexcitability hypothesis advocates enhancing neural activity within the lesioned affected hemisphere [26]. Clinical investigations have obtained conflicting results among patients with different stroke phases and varying severities of neurological deficits, and no consistent consensus has been reached on the optimal intervention strategies so far [24,40].

Investigations have predominantly focused on single biomarkers, and studies exploring combinations of multiple biomarkers—for example, through integrating imaging and molecular/genetic indicators or combining electrophysiological parameters with molecular, genetic, or imaging biomarkers—remain scarce. Future research could develop multimodal biomarker panels for post-stroke neuroplasticity to improve the accuracy of predicted neuroplastic changes after ischemic stroke. In this scoping review, we found that most correlation studies are cross-sectional, lacking long-term longitudinal predictive evidence, and multiple confounding factors have not been effectively controlled.

Regarding rehabilitation therapies and interventions, intense debate persists concerning the optimal time window. Conventional studies hold that the subacute phase constitutes the optimal time window to induce and enhance adaptive neuroplasticity [8,45,109]. Accumulating evidence confirms that patients with chronic stroke still retain substantial neuroplastic potential [23,25,30]. In research on interventions, non-invasive brain stimulation techniques have been widely investigated [23,105,107]. However, discrepancies in stimulation polarity, targeted sites, and sham control protocols have resulted in inconsistent therapeutic effects in relevant clinical trials.

Various targeted drugs and stem cell therapies have achieved prominent efficacy in preclinical experiments. However, most of them have not been applied clinically or have failed to obtain stable positive results in clinical trials, creating an obvious translational gap between basic research and clinical practice. In addition, there are inconsistent research standards for emerging and conventional rehabilitation therapies, including acupuncture, virtual reality, brain–computer interfaces, and aerobic exercise. The underlying mechanisms by which these interventions regulate neuroplasticity remain to be further verified. Compared with single therapeutic approaches such as isolated rehabilitation training and drug intervention, combined treatment regimens integrating rehabilitative exercise, neuromodulation techniques, and drug interventions may exert synergistic therapeutic effects through mechanistic collaboration. Such multimodal interventions can effectively facilitate adaptive neural remodeling and curb the progression of maladaptive plasticity, yet relevant studies on combined multimodal interventions remain insufficient at present.

### 4.2. Research Gaps and Future Perspectives

Recovery after post-ischemic stroke brain injury is influenced by multiple factors, and overall recovery depends on the interaction of genetic factors, pathophysiological mechanisms, environmental conditions, therapeutic interventions, and other multi-dimensional factors, which also makes the prediction of stroke prognosis very challenging. Contemporary investigations are more focused on neurovascular aspects but research on post-ischemic stroke plasticity mechanisms has challenged traditional viewpoints. Discoveries in mechanisms such as synaptic remodeling, axonal sprouting, and neurogenesis have provided scientific evidence for determining the timing of rehabilitation interventions. In particular, the acute to subacute phase (within 3 months post-stroke) has been established as the golden window for neuroplasticity regulation intervention. At the same time, this research has driven innovations in rehabilitation technology, offering more options to improve the prognosis of stroke patients and significantly mitigating the limitations of traditional treatment methods. These findings have prompted the rehabilitation concept to shift from functional compensation to neuroplasticity, establishing the modern rehabilitation principles of early intervention and high-intensity repetitive training. However, there are still significant challenges in establishing a biomarker system for quantitatively assessing neural plasticity. Studies on the plasticity mechanisms of ischemic stroke have mostly been conducted in animal models or in vitro experiments, while studies on stroke patients remain extremely scarce. It is still unclear whether the mechanisms identified in animal and in vitro studies are applicable to human stroke patients, and further research is needed. Through this comprehensive review, we found that clinical studies investigating the mechanisms underlying neuroplasticity after stroke are relatively scarce, whereas studies on related biomarkers and interventions are more numerous.

In addition to conventional pharmacological treatments, drugs targeting genes associated with neural plasticity markers can provide new approaches for the precision treatment of ischemic stroke in the future. However, there are still numerous challenges in this field, and it remains uncertain whether the in vitro findings can be applied clinically. At the same time, how to enhance endogenous plasticity via non-invasive neuromodulation techniques will become a research hotspot in translational medicine. With the development of cutting-edge technologies such as brain–computer interfaces, stroke rehabilitation is expected to enter a new stage of ‘precise regulation of neural plasticity,’ and finally achieve a leap from basic research to clinical application, which could contribute to improving the prognosis of stroke patients.

At present, a variety of potential biomarkers associated with post-stroke neuroplasticity and novel therapeutic strategies have been gradually explored, yet their large-scale clinical promotion and routine clinical application still face numerous practical constraints. The main restrictive factors are comprehensively analyzed as follows. Firstly, substantial differences exist in research methodologies across relevant studies. Different detection techniques, experimental designs, outcome indicators, and evaluation standards adopted by distinct research teams easily lead to inconsistent research conclusions, which impede the formation of a unified consensus and standardized clinical application criteria. Secondly, most existing relevant studies have relatively small sample sizes, and single-center research accounts for the majority. These limitations fail to effectively reduce selection bias and individual heterogeneity, leaving research findings lacking sufficient population representativeness and stable extrapolation value. Thirdly, there is still a lack of unified and standardized research protocols in this field. No consistent norms have been established for subject inclusion criteria, intervention courses, detection time points, and result judgment principles, leading to poor research repeatability and making it difficult to develop operable clinical application specifications. Fourthly, high-quality multi-center clinical validation is seriously insufficient. Most current evidence comes from retrospective analyses or small-sample exploratory trials, while large-sample, multi-center studies with long-term follow-up verification remain scarce. Without adequate multi-center evidence support, it is difficult to fully confirm the long-term safety, actual efficacy, and scope of applicable populations for these biomarkers and new therapies, which greatly restricts the transformation of basic research achievements into mature clinical practice.

### 4.3. Strengths and Limitations

Several limitations of this scoping review should be acknowledged. First, only English-language literature published in the last five years were included, which may have resulted in incomplete literature retrieval due to the relatively narrow time span and language bias, and potentially excluding relevant valuable findings. Second, some of the included studies had small sample sizes and relatively short follow-up durations, limiting the ability to draw robust conclusions about long-term neuroplastic changes. Finally, animal and human studies were not subjected to meta-analysis, which prevents a direct synthesis of the preclinical and clinical evidence on post-stroke neuroplastic mechanisms.

## 5. Conclusions and Outlook

In summary, post-ischemic stroke neuroplasticity exhibits clear temporal dynamics across the acute, subacute, and chronic stages. Molecular, electrophysiological, and imaging biomarkers enable dynamic monitoring of these plastic processes. Interventions such as rehabilitation, neuromodulation, and pharmacotherapy hold promise for promoting adaptive remodeling while suppressing maladaptive plasticity. Future research should prioritize the development of stage-specific biomarkers and the investigation of individualized, combinatorial therapeutic strategies to optimize functional recovery. Although the reviewed studies have provided some novel insights, most molecular biomarkers and emerging therapeutic interventions still require further validation through large-scale, multicenter, higher quality prospective clinical studies before they can be safely and effectively incorporated into routine clinical practice.

## Figures and Tables

**Table 1:** Characteristics of studies included in the review.

Author and Year	Country	Study Design	Total Sample Size	Participant Characteristics	Research Domain	Key Findings
Altintas et al. (2022) [5]	Turkey	Retrospective case series	90 patients	Acute ischemic stroke	Biomarkers	Serum TRAIL and adropin levels were associated with poor clinical outcomes
Zhang et al. (2024) [6]	China	Observational study	25 patients	Stroke	Mechanism	IL-CST showed aggravation followed by improvement from around the lesion to the distal end
Sun et al. (2023) [7]	China	Randomized controlled trial	98 patients	Acute ischemic stroke	Mechanism + Intervention	A computer-intelligent segmentation model LT-RCNN could accurately locate and segment AIS lesions and early rehabilitation training could change the expression of inflammatory factors
Liu et al. (2024) [19]	China	Observational study	25 patients	Stroke	Mechanism + Biomarkers	Highlighted the potential of muscle synergy plasticity as a valuable tool for monitoring rehabilitation progress
Lin et al. (2026) [21]	China	Case–control	27 stroke patients	Stroke	Biomarkers	Developed EEG-fNIRS multilayer brain network analysis method
Yue et al. (2025) [22]	China	Retrospective study	20 patients	Acute ischemic stroke	Biomarkers	Highlighted the protective roles of moderate immune-inflammatory activation
Carlos et al. (2025) [23]	Brazil	Single-masked	20 subjects	Chronic stroke	Intervention	Anodal tDCS combined with XR therapy seems to enhance neuroplasticity by modulating high-frequency power and connectivity
Hill et al. (2023) [24]	Australia	Randomized controlled trial	33 patients	Stroke	Intervention	Moderate-intensity cycling may enhance neuroplasticity in people with stroke
Kim et al. (2025) [25]	Korea	Randomized controlled trial	25 patients	Chronic stroke	Intervention	Developed brain–computer interface training with MI-contingent feedback approach
Guo et al. (2025) [26]	China	Randomized controlled trial	40 patients	Subacute ischemic stroke	Intervention	Concurrent tDCS during VR-based robotic intervention can effectively enhance upper limb function and promote activation of ipsilesional M1 and contralesional PFC
Gerloff et al. (2022) [20]	Germany, Austria, USA	Randomized, double-blind, placebo-controlled trial	120 patients	Subacute ischemic stroke	Intervention	Provides clinically relevant information on the topic of adjuvant non-invasive brain stimulation after stroke to enhance motor recovery
Umar et al. (2025) [27]	Malaysia	Randomized controlled trial	69 patients	Stroke recovery	Intervention	The combination of TRE and tDCS may be a promising approach to enhance trunk control and mobility in post-stroke patients
Sihvonen et al. (2022) [28]	Finland	Secondary analysis of a randomized controlled trial	38 patients	Stroke	Intervention	Showed that listening to music, either vocal or instrumental, promotes wide-spread structural connectivity changes in the post-stroke brain
Song et al. (2025) [29]	China	Randomized controlled trial	50 patients	Stroke	Intervention	Acupuncture combined with the use of an upper limb rehabilitation robot can effectively improve upper limb function and neural remodeling
Gangemi et al. (2023) [30]	Italy	Randomized clinical trial	30 patients	Chronic stroke	Intervention	Results indicate that a VR-based rehabilitation approach has potential in promoting neuroplastic changes

## Data Availability

No new data were created or analyzed in this study.

## Supplementary Materials

The following supporting information can be downloaded at: https://www.mdpi.com/article/10.3390/brainsci16080784/s1, Table S1: Preferred Reporting Items for Systematic reviews and Meta-Analyses extension for Scoping Reviews (PRISMA-ScR) Checklist; Table S2: Full Search Strategies for PubMed; Table S3: Animal and in vitro studies on neural plasticity after ischemic stroke; Table S4: Summary of reviews on neural plasticity after ischemic stroke.

## Abbreviations

The following abbreviations are used in this manuscript:

DALYs	Disability-adjusted life years
PRISMA-ScR	Preferred Reporting Items for Systematic Reviews and Meta-Analyses Extension for Scoping Reviews
MeSHs	Medical Subject Headings
SVZ	Subventricular zone
DG	Dentate gyrus
BDNF	Brain-derived neurotrophic factor
NSC	Neural stem cell
GFAP	Glial fibrillary acidic protein
SDF1α	Stromal cell-derived factor-1α
rs-fMRI	Resting-state functional magnetic resonance imaging
TMS	Transcranial magnetic stimulation
MEG	Magnetoencephalography
EEG	Electroencephalography
tDCS	Transcranial direct current stimulation
NGF	Nerve growth factor
NT-3	Neurotrophin-3
NT-4	Neurotrophin-4
CNS	Central nervous system
