# Supplementary material for: Mechanisms, Biomarkers, and Therapeutic Interventions of Neuroplasticity After Ischemic Stroke—A Scoping Review
Source: Brain Sci. 2026 Jul 25;16(8):784. doi: 10.3390/brainsci16080784 (PMC13510713; doi:10.3390/brainsci16080784)
Supplement: Supplementary file 1 [file brainsci-16-00784-s001.zip › Supplementary Table_S1_PRISMA-ScR-Fillable-Checklist_brainsci-4412299.pdf]

Table S1 Preferred Reporting Items for Systematic reviews and Meta-Analyses extension for Scoping Reviews (PRISMA-ScR) Checklist

SECTION	ITEM	PRISMA-ScR CHECKLIST ITEM	REPORTED ON PAGE #
**TITLE**			
Title	1	Identify the report as a scoping review.	**Page 1 (Title):** The subtitle includes "A Scoping Review"
**ABSTRACT**			
Structured summary	2	Provide a structured summary that includes (as applicable): background, objectives, eligibility criteria, sources of evidence, charting methods, results, and conclusions that relate to the review questions and objectives.	**Page 1-2 (Abstract):** Contains structured sections: Background,Methods, Results, Conclusions.
**INTRODUCTION**			
Rationale	3	Describe the rationale for the review in the context of what is already known. Explain why the review questions/objectives lend themselves to a scoping review approach.	**Page 2-3 (Introduction) :** Describes the fragmented evidence for post-ischemic stroke neuroplasticity. Therefore, a scoping review was conducted to comprehensively...
Objectives	4	Provide an explicit statement of the questions and objectives being addressed with reference to their key elements (e.g., population or participants, concepts, and context) or other relevant key elements used to conceptualize the review questions and/or objectives.	**Page 3 (Aim and Objectives):** To systematically map and rigorously evaluate the existing evidence on the mechanisms, biomarkers, and interventions for neuroplasticity after ischemic stroke.
**METHODS**			
Protocol and registration	5	Indicate whether a review protocol exists; state if and where it can be accessed (e.g., a Web address); and if available, provide registration information, including the registration number.	**Page 3 (Methods, first paragraph):** This scoping review was conducted following the PRISMA-ScR,The protocol has been registered with the Open Science Framework (https://osf.io/78zxd).
Eligibility criteria	6	Specify characteristics of the sources of evidence used as eligibility criteria (e.g., years considered, language, and publication status), and provide a rationale.	**Page 3-4 (Eligibility Criteria):** Uses PCC framework (Population, Concept, Context). Specifies ischemic stroke stage, aged, mechanism, biomarkers, interventions,date range (Jan 2021- Jan 2026), English language, and rationale for exclusions.
Information sources*	7	Describe all information sources in the search (e.g., databases with dates of coverage and contact with authors to identify additional sources), as well as the date the most recent search was executed.	**Page 4 (2.2. Information Sources...):** Lists three databases and states search covered studies "from January 31, 2021 to January

St. Michael's
Inspired Care.
Inspiring Science.

SECTION	ITEM	PRISMA-ScR CHECKLIST ITEM	REPORTED ON PAGE #
			31, 2026
Search	8	Present the full electronic search strategy for at least 1 database, including any limits used, such that it could be repeated.	**Page 4 (2.2. Information Sources...):** Describes search keywords and MeSH.The full electronic search strategy for PubMed is provided in the supplementary material.
Selection of sources of evidence†	9	State the process for selecting sources of evidence (i.e., screening and eligibility) included in the scoping review.	**Page 4-5 (2.3 Selection of Sources of Evidence):** Describes steps: duplicate removal, title/abstract screening by two researchers, full-text assessment.
Data charting process‡	10	Describe the methods of charting data from the included sources of evidence (e.g., calibrated forms or forms that have been tested by the team before their use, and whether data charting was done independently or in duplicate) and any processes for obtaining and confirming data from investigators.	**Page 5 (2.4 Data Extraction):**A standardized extraction form was developed, and key extracted variables are mentioned. Duplicate charting by two independent reviewers was conducted. The form is included in the Supplementary Material.
Data items	11	List and define all variables for which data were sought and any assumptions and simplifications made.	**Page 5 (2.4 Data Extraction):** Lists key variables:research subjects, characteristics, sample size, research domain,and key findings.
Critical appraisal of individual sources of evidence§	12	If done, provide a rationale for conducting a critical appraisal of included sources of evidence; describe the methods used and how this information was used in any data synthesis (if appropriate).	**Page 5 (2.5 Critical appraisal of...):** We did not conduct a formal critical appraisal of the included studies. Detailed justifications are provided in the Methods section.
Synthesis of results	13	Describe the methods of handling and summarizing the data that were charted.	**Page 5** (**2.6 Data Analysis and Synthesis):** Describes descriptive numerical summary and narrative synthesis, with data tabulation.
**RESULTS**			
Selection of sources of evidence	14	Give numbers of sources of evidence screened, assessed for eligibility, and included in the review, with reasons for exclusions at each stage, ideally using a flow diagram.	**Page 6 (3.1 Study Selection):** Provides numbers: 974 records, 513 duplicates removed, 461 screened, 412 excluded, 49 full-text assessed, 33 excluded, 16 included. **Figure1** is the PRISMA flow diagram.

SECTION	ITEM	PRISMA-ScR CHECKLIST ITEM	REPORTED ON PAGE #
Characteristics of sources of evidence	15	For each source of evidence, present characteristics for which data were charted and provide the citations.	**Page 6-7 (3.2. Characteristics of... & Table 1):** Table 1 summarizes key characteristics (author and year, country, study design, sample size, participant characteristics, research domain and key findings) of the 16 included studies, with citations.
Critical appraisal within sources of evidence	16	If done, present data on critical appraisal of included sources of evidence (see item 12).	**N/A (Not formally conducted).** No critical appraisal of the internal data within each individual included study was performed in this scoping review.
Results of individual sources of evidence	17	For each included source of evidence, present the relevant data that were charted that relate to the review questions and objectives.	**Page 8-12 (3.3.Mechanisms of ..., 3.4. Biomarkers of ..., 3.5. Pharmacological and...):** Relevant extracted data corresponding to the research questions and review objectives from all eligible studies were systematically sorted out and clearly presented in standardized data extraction tables.
Synthesis of results	18	Summarize and/or present the charting results as they relate to the review questions and objectives.	**Page 12-13 (3.6. Comparison..., 3.7. Distribution...):** A narrative summary of the core findings was conducted in line with the review objectives, including analysis of research heterogeneity, trends of research outcomes, existing research limitations and unaddressed research gaps in the field of neuroplasticity after ischemic stroke.
**DISCUSSION**			
Summary of evidence	19	Summarize the main results (including an overview of concepts, themes, and types of evidence available), link to the review questions and objectives, and consider the relevance to key groups.	**Page 13-16 (4.1. Main Findings..., 4.2. Research gaps...）:** This section summarizes the core main findings that existing relevant studies are one-sided and fragmented. Combined with the review objectives, it elaborates research designs, research outcomes and current research gaps. Furthermore, it discusses the practical implications of the

St. Michael's
Inspired Care.
Inspiring Science.

SECTION	ITEM	PRISMA-ScR CHECKLIST ITEM	REPORTED ON PAGE #
			present evidence, emphasizes the urgent need to carry out in-depth mechanism research among stroke populations, and proposes the necessity of developing multi-modal combined research.
Limitations	20	Discuss the limitations of the scoping review process.	**Page 16 (4.3. Strengths and Limitations):** Lists limitations: narrow time span, the quality of included studies varies greatly, and exclusion of non-English studies.
Conclusions	21	Provide a general interpretation of the results with respect to the review questions and objectives, as well as potential implications and/or next steps.	**Page 16 (5. Conclusions & Outlook):** It interprets that the existing evidence on mechanisms, biomarkers and interventions is still in the preliminary exploration and preclinical research stage, and puts forward specific implications for future research and clinical practice.
**FUNDING**			
Funding	22	Describe sources of funding for the included sources of evidence, as well as sources of funding for the scoping review. Describe the role of the funders of the scoping review.	**Page 16 (Funding):** This research was funded by the National Natural Science Foundation of China (grant number 82201622).

JBI = Joanna Briggs Institute; PRISMA-ScR = Preferred Reporting Items for Systematic reviews and Meta-Analyses extension for Scoping Reviews.
* Where *sources of evidence* (see second footnote) are compiled from, such as bibliographic databases, social media platforms, and Web sites.
† A more inclusive/heterogeneous term used to account for the different types of evidence or data sources (e.g., quantitative and/or qualitative research, expert opinion, and policy documents) that may be eligible in a scoping review as opposed to only studies. This is not to be confused with *information sources* (see first footnote).
‡ The frameworks by Arksey and O'Malley (6) and Levac and colleagues (7) and the JBI guidance (4, 5) refer to the process of data extraction in a scoping review as data charting.
§ The process of systematically examining research evidence to assess its validity, results, and relevance before using it to inform a decision. This term is used for items 12 and 19 instead of "risk of bias" (which is more applicable to systematic reviews of interventions) to include and acknowledge the various sources of evidence that may be used in a scoping review (e.g., quantitative and/or qualitative research, expert opinion, and policy document).

From: Tricco AC, Lillie E, Zarin W, O'Brien KK, Colquhoun H, Levac D, et al. PRISMA Extension for Scoping Reviews (PRISMAScR): Checklist and Explanation. Ann Intern Med. 2018;169:467–473. doi: 10.7326/M18-0850.
