# Supplementary material for: Mechanisms, Biomarkers, and Therapeutic Interventions of Neuroplasticity After Ischemic Stroke—A Scoping Review
Source: Brain Sci. 2026 Jul 25;16(8):784. doi: 10.3390/brainsci16080784 (PMC13510713; doi:10.3390/brainsci16080784)
Supplement: Supplementary file 1 [file brainsci-16-00784-s001.zip › Supplementary Table_S2.pdf]

**Table S2** Full Search Strategies for PubMed

| Database | | | SEARCH STRATEGIE | RESULT |
|---|---|---|---|---|
| PubMed | #1 | POPULATION | (cerebral infarction[MeSH Terms] OR ischemic stroke[MeSH Terms] OR cerebral infarct*[Title/Abstract] OR brain infarction[Title/Abstract] OR focal cerebral ischemia[Title/Abstract]) | |
| | #2 | CONCEPT | (neuroplasticity[MeSH Terms] OR neuronal plasticity[Title/Abstract] OR brain plasticity[Title/Abstract] OR synaptic plasticity[Title/Abstract] OR mechanism[Title/Abstract] OR pathogenesis[Title/Abstract] OR biomarker*[Title/Abstract] OR biological marker*[Title/Abstract] OR intervention*[Title/Abstract] OR therapy[Title/Abstract] OR rehabilitation intervention[Title/Abstract] OR drug intervention[Title/Abstract]) | |
| | #3 | CONTEXT | (acute Disease[MeSH Terms] OR Subacute Disease[MeSH Terms] OR Recovery[MeSH Terms] OR subacute[Title/Abstract] OR recovery phase[Title/Abstract] OR post-stroke recovery[Title/Abstract] OR rehabilitation phase[Title/Abstract]) | |
| | | | #1 AND #2 AND #3 AND ("2021/01/31"[Date - Publication] : "2026/01/31"[Date - Publication]) | 284 |
