# Supplementary material for: Mechanisms, Biomarkers, and Therapeutic Interventions of Neuroplasticity After Ischemic Stroke—A Scoping Review
Source: Brain Sci. 2026 Jul 25;16(8):784. doi: 10.3390/brainsci16080784 (PMC13510713; doi:10.3390/brainsci16080784)
Supplement: Supplementary file 1 [file brainsci-16-00784-s001.zip › Supplementary Table_S3.pdf]

Table S3. Animal and in vitro studies on neural plasticity after ischemic stroke.

Author and Year	Country	Experimental model source	Research Domain	Key Findings
Beker et al. (2025) [33]	Turkey	Adult male C57BL/6j mice (8–12 weeks)	Intervention + Mechanism	1.Melatonin enhances neurogenesis and neuroplasticity in long-term recovery following cerebral ischemia in mice.<br>2.Studies have found that melatonin regulates the expression of various membrane-localized proteins associated with neural plasticity, including ephrin B1, ephrin B2, brevican and versican, thereby inducing structural remodeling of cells and the extracellular matrix.
Shi et al. (2026) [36]	China	Male SD rats (8 weeks old, 220 – 250 g)	Mechanism	AAV-sh. ROCK2 increased neurite outgrowth and facilitated synaptogenesis in vivo.
Pirscoveanu et al. (2025) [37]	Germany	Young male SD rats (3 - 4 months old) and old rats	Mechanism	1. Gene variance in juvenile brains predominantly reflected processes associated with brain plasticity (e.g., Dcx, Tubb2b, Dok4, Dpysl5) and cell proliferation (e.g., Bex4).<br>2. Immune genes that facilitate synaptic plasticity during early postnatal brain development—through processes such as pruning and sprouting to establish new connections in response to external stimuli.
Yan et al. (2023) [38]	China	Aged (18 – 20 months) male SD rats (600 – 800 g)	Intervention + Mechanism	1. Environmental enrichment enhanced the proliferation of newborn neurons in the SVZ, as well as increased the long-term survival of newborn neurons.<br>2. Environmental enrichment also exerted effects on inflammation after stroke.
DeBoer et al. (2021) [39]	United States	Male Mice	Intervention	Poststroke Cerebrolysin administration leads to recovery of motor function independent of rehabilitative training without a protective effect on stroke volume.
Matsui et al. (2022) [50]	Japan	In vitro experiments	Mechanism	IL4 Stimulated Macrophages Promote Axon Regeneration after Peripheral Nerve Injury by Secreting uPA to Stimulate uPAR Up-regulated in Injured Axons.
Cheng et al. (2025) [65]	China	Male SD rats (8 weeks old, 220 – 240 g)	Intervention + Mechanism	Yindan Xinnaotong Soft Capsule promoted post-ischemic stroke recovery by promoting CREB/BDNF-mediated synaptic plasticity and CREB/VEGFA-mediated angiogenesis.
Kaur et al. (2023) [66]	India	Adult female SD rats (220–250 g)	Mechanism	1. Post-stroke IA MSC administration significantly increased BDNF expression and decreased ER stress markers expression

				at day 1 post-stroke.<br>2. A gradual rise in the expression of growth-associated protein-43 (GAP43) and spinophilin was observed at 7, 14-, and 28-days post-stroke, indicating an increase in neuronal remodeling toward functional restoration.
Yang et al. (2025) [76]	China	Adult male C57BL/6J mice (8 – 10 weeks old, 24.0 – 26.0 g)	Mechanism	NAT10 regulates astrocyte autophagy by targeting Timp1 ac4C after ischemic stroke.
Yang et al. (2025) [79]	China	GEO database model mice exposed to MCAO	Mechanism	There is a close association of CCR5 and CXCR4 with post-ischemic stroke neuroplasticity.
Mainali et al. (2025) [81]	United States	In vitro experiments + review	Mechanism + Biomarker Mechanism + Biomarker	Within the first 6 h of symptom onset, three specific miRNAs (miR-7-5p, miR-140-5p, and miR-210-3p) exhibited significant differential expression compared to other time points and healthy controls.
Cai et al. (2021) [85]	China	SD rats (8 weeks old, 150-200 g)	Mechanism	NFF promoted recovery of neurological function through BDNF signaling pathways.<br>NFF increased the expression of SYN1 in rats and cultured neurons.
Yang et al. (2025) [86]	China	In vitro experiments + Modified bilateral carotid artery ligation rats.	Mechanism	Zhongfeng Xingnao Liquid significantly increased the B-cell lymphoma 2/Bcl2-associated X ratio, reduced terminal deoxynucleotidyl transferase-mediated dUTP nick-end-labeling cells, and markedly improved cognition, synaptic plasticity, and neuronal function in the hippocampus and cortex.
Wang et al. (2024) [93]	China	Adult male C57/BL6 mice (8–10 weeks old, 20.0 – 25.0 g)	Mechanism	ANXA6 enhances synaptic plasticity and protects against transient cerebral ischemia.
Yamaguchi et al. (2021) [95]	Japan	C.B-17/ICR-Jcl mice	Intervention + Mechanism	Voluntary Running Exercise after Focal Cerebral Ischemia Ameliorates Dendritic Spine Loss and Promotes Functional Recovery.
Olmedo-Córdoba et al. (2025) [105]	Spain	Male Wistar rats from Envigo (Barcelona, Spain), (250 – 275 g)	Intervention + Mechanism	tDCS led to a significant increase in different neuroplasticity markers, such as Htr2a, Grin1, Bdnf, Ngf, and Scn2a in the FC of compulsive rats compared to sham treatment.
Huang et al. (2025) [107]	China	Male SD rats weighing (250-280 g)	Intervention + Mechanism	Dual-tDCS improved motor function and infarct volume, inhibited neuronal apoptosis, promoted the expression of

				neurotrophins (BDNF and NGF), CD31, and VEGF, and suppressed the inflammatory reaction after MCAO via the cGAS-STING pathway.
Wang et al. (2024) [109]	China	C57BL/6 male mice (8 weeks old, 20 – 25 g)	Intervention + Mechanism	Elucidate for the first time the role of 2-acetylacteoside in enhancing neurological recovery, primarily by promoting neurogenesis via Akt activation following ischemic brain injury.
