# Supplementary material for: Mechanisms, Biomarkers, and Therapeutic Interventions of Neuroplasticity After Ischemic Stroke—A Scoping Review
Source: Brain Sci. 2026 Jul 25;16(8):784. doi: 10.3390/brainsci16080784 (PMC13510713; doi:10.3390/brainsci16080784)
Supplement: Supplementary file 1 [file brainsci-16-00784-s001.zip › Supplementary Table_S4.pdf]

Table S4. Summary of reviews on neural plasticity after ischemic stroke.

Author & Year	Country	Review type	Research Domain	Key Findings
Qiao et al. (2023) [4]	China	Narrative Review	Interventions + Mechanisms	1. Mechanism: remodeling of dendrites and dendritic spines, myelin regeneration, and neurogenesis.<br>2. Mechanisms of microglial activation, polarization, and phagocytosis.<br>3. Therapeutic Intervention Targeting Microglia in Stroke Rehabilitation.
Yu et al. (2021) [8]	China	Narrative Review	Mechanisms	Focus on the crosstalk between microglial activation and endogenous neuroplasticity, with a special focus on the plastic alterations in the whole-brain network and their implications for structural and functional restoration after stroke.
Qi et al. (2022) [9]	United States	Narrative Review	Mechanisms	Review the molecular mechanisms of synaptogenesis by going over the studies on the identification of molecular components in synapses and their functions in synaptogenesis.
Ramírez-Expósito et al. (2026) [10]	Spain	Narrative Review	Mechanisms	This review has described the molecular mechanisms that control presynaptic neurotransmitter release, postsynaptic signal transduction, and the processes underlying synaptic plasticity, formation, maturation, and elimination.
Jing et al. (2023) [11]	China	Narrative Review	Mechanisms	This review summarizes the latest evidence of astrocyte involvement in PSD from human and animal studies, focusing on the alterations of astrocyte markers and functions in relation to monoamine neurotransmitters, inflammatory cytokines, brain-derived neurotrophic factor, and glutamate excitotoxicity.
Milani et al. (2022) [12]	Italy	Scoping Review	Biomarkers	Data suggest that the most promising EEG quantifiers for predicting post-stroke motor outcome are event-related measures.
Florijn et al. (2023) [13]	Netherlands	Systematic Review and Meta-Analysis	Biomarkers	1. Circulating microRNAs (< 24 h) and proteins (< 4.5 h) perform equally well in ischemic stroke diagnosis.<br>2. GFAP differentiates stroke subtypes, while a biomarker panel of GFAP and UCH-L1 improved the sensitivity and specificity of UCH-L1 alone to differentiate stroke.
Qin et al. (2022) [14]	China	Literature Review	Interventions	The impact of acupuncture on neuroplasticity after ischemic stroke: Acupuncture promotes the replacement of functional cells that have died after stroke by stimulating nerve cell regeneration, laying the structural foundation for the establishment of contacts and synapses between cells by promoting the growth of new nerve processes or the regeneration of damaged axons, and thus ultimately promoting information

				transfer by enhancing structural connections.
Saway et al. (2024) [15]	USA.	Narrative Review	Interventions	Review invasive neuromodulation techniques to augment the effects of conventional rehabilitation methods are described, including vagus nerve stimulation , deep brain stimulation, and brain-computer interfaces .
Wang et al. (2022) [16]	China	Narrative Review	Interventions + Mechanisms	Focus on the role and mechanisms of microglia-mediated neuroinflammation and neuroplasticity after ischemia and relevant potential microglia-based interventions for stroke therapy.
Zhang et al. (2021) [17]	China	Scoping Review	Interventions + Mechanisms	Acupuncture therapy can modulate the function and structural plasticity of post-stroke, which may be linked to the mechanism of acupuncture.
Mesquida-Veny et al. (2021) [71]	Spain	Narrative Review	Mechanisms	Review the complexity of macrophages and microglia after neuronal injury and their roles in regeneration, as well as the underlying molecular mechanisms.
